# Design and Fabrication of 3D printed Scaffolds with a Mechanical Strength Comparable to Cortical Bone to Repair Large Bone Defects

**DOI:** 10.1038/srep19468

**Published:** 2016-01-19

**Authors:** Seyed-Iman Roohani-Esfahani, Peter Newman, Hala Zreiqat

**Affiliations:** 1 Biomaterials and Tissue Engineering Research Unit, School of Aeronautical Mechanical and Mechatronics Engineering, University of Sydney, 2006, NSW, Australia

## Abstract

A challenge in regenerating large bone defects under load is to create scaffolds with large and interconnected pores while providing a compressive strength comparable to cortical bone (100–150 MPa). Here we design a novel hexagonal architecture for a glass-ceramic scaffold to fabricate an anisotropic, highly porous three dimensional scaffolds with a compressive strength of 110 MPa. Scaffolds with hexagonal design demonstrated a high fatigue resistance (1,000,000 cycles at 1–10 MPa compressive cyclic load), failure reliability and flexural strength (30 MPa) compared with those for conventional architecture. The obtained strength is 150 times greater than values reported for polymeric and composite scaffolds and 5 times greater than reported values for ceramic and glass scaffolds at similar porosity. These scaffolds open avenues for treatment of load bearing bone defects in orthopaedic, dental and maxillofacial applications.

The ability of bone to self-repair after fracture is limited according to the extent of the damage; small fractures are usually able to heal perfectly, but larger fractures, known as segmental bone defects (SBDs), can leave permanent damage^1^,^2^. The common treatment for SBDs is an autologous bone graft, which involves harvesting of non-essential bone, for example the iliac crest. However, this method is limited by the availability of bone, donor site morbidity, risk of infection and geometric mismatch between the harvested bone and the defect site, which can result in voids and poor integration^1^,^3^. Repairing SBDs remains a major surgical challenge and suboptimal outcomes can have significant socio-economic repercussions and negatively affect quality of life^3^,^4^. Over the past 30 years, a wide range of innovative synthetic materials have been developed to overcome the problems associated with autologous bone grafts. These materials include bioceramics (typically calcium phosphates (CaPs) and bioactive glasses), polymers (naturally derived such as collagen-I and synthetic such as polycaprolactone (PCL)) and hybrid materials (a mixture of bioceramics and polymers)^5^,^6^,^7^,^8^. None of these materials have had the strength required to withstand static and cyclic loads *in vivo* whilst maintaining sufficiently high porosity to facilitate bone ingrowth, vascularisation and the transport of nutrients. In order to meet these needs, the ideal scaffold requires porosity between 60% and 90% with an average pore size of >150 μm and compressive strength comparable to that of cortical bone, which is in the range of 100 to 150 MPa along the long axis^2^,^9^,^10^. Weakness associated with current highly porous scaffolds continues to fuel the demand for a high strength bone scaffold for treatment of the SBDs. Recently Eqtesadi *et al*. utilised the robocasting technique to fabricate 13–93 bioactive glass scaffolds with average strut thickness of 274 μm, pore size of ~230 μm and porosity of 51%. The scaffolds showed a brittle behaviour with a compressive strength of 86 MPa and a bending strength of 15 MPa. They infiltrated the scaffolds with a tough polymer (PCL) to address the brittleness and further improve the strength of scaffolds. Although toughness of scaffolds significantly improved, their compressive and bending strength did not significantly change^11^. Dai *et al*. developed a calcium silicate scaffold with a compressive strength range of 28.1 to 10.3 MPa following the porosity from 53% to 71%^12^. Feng *et al*. used selective laser sintering method to fabricate strong akermanite (Ca_2_MgSi_2_O_7_) scaffolds reinforced with nano-titania particles. They reported a maximum compressive strength of 23 MPa for scaffolds with ~58% porosity after addition of 5 wt.% nano-titania^13^. Robert *et al*. developed a new method to enhance mechanical strength of collagen scaffolds to be used as a synthetic bone graft under load. They prepared hydroxyapatite(HA) reinforced collagen scaffolds by compression molding of HA reinforcements and paraffin microspheres within a suspension of concentrated collage fibrils then crosslinking the collagen matrix and leaching the paraffin porogen. The final scaffold (porosity of 85% and pore size of 350 μm) exhibited a compressive modulus and strength of ~1 MPa and 15 KPa, respectively^14^. Baino *et al*. fabricated a glass-ceramic scaffold to repair large defects in load-bearing bones by sponge template method^15^. Their scaffold had a total porosity of 56% and the pore sizes ranged within 100–500 μm. When tested under compression, the scaffolds showed strength of 18 MPa, an elastic modulus of 380 MPa and Weibull modulus of 4. Chen *et al*. developed titanium scaffolds with a range of porosity between 28% and 50% by a centrifugal granulation technology for the repair of load-bearing bone defects. The compressive strength of scaffolds was reported to be between 83 and 109 MPa, respectively^16^. Feng *et al*. incorporated HA whiskers into calcium silicate matrix to improve the strength of calcium silicate based scaffolds. They showed that compressive strength of scaffolds with ~45% porosity increased from 15 MPa to 27 MPa by addition of 20 wt.% whiskers^17^. Flauder *et al*. developed anisotropic scaffolds with lamellar structure and adjustable porosities from 49 to 82% by directional solidification of water-based β-tricalcium phosphate (β-TCP) suspensions. They demonstrated that correlated compressive strengths of the scaffolds reached from 0.4 MPa to 40 MPa^18^. McNamara *et al*. developed a new technique to fabricate HA scaffolds for load bearing applications. They used naturally derived silk from Bombyx mori for ceramic grain consolidation during green body formation, and later as a sacrificial polymer to impart porosity during sintering. This technique allowed preparation of HA scaffolds that exhibited a compressive strength of 8.7 MPa at porosity of 63%. They also obtained compressive strengths up to 152 MPa for scaffolds with porosity of ~20%^19^. Rakovsky *et al*. combined β-TCP and polylactic acid(PLA) to fabricate strong composite scaffolds by salt leaching technique^20^. Composite scaffolds with 50% porosity and large pore size (300–420 μm) showed a compressive strength of ~5 MPa falling within the range of trabecular bone. Xu *et al*. developed a ceramic (nagelschimdtite, Ca_7_Si_2_P_2_O_16_) scaffold with average porosity of 55% by 3-D plotting method. They reported that scaffolds had a compressive strength of ~15 MPa that was insufficient for load-bearing applications in bone regeneration^21^. Zhou *et al*. aimed to improve mechanical strength of CaP based scaffolds by compositing calcium phosphate with collagen. Scaffolds were made by foaming method and then immerged into collagen solution. They obtained the highest compressive strength of 7 MPa for a composite scaffold with 75% porosity that was approximately 3 times more than that for the original ceramic scaffold^22^. Houmard *et al*. fabricated HA/β-TCP(20 wt.%/80 wt.%) scaffolds at various porosities using robocasting (25-80%)^23^. They reported that compressive strength of the fabricated scaffolds varied between ~3 and ~50 MPa. Huang *et al*. introduced a motor assisted micro-syringe technique to fabricate HA/β-TCP scaffolds with homogeneous and interconnected pores in sizes ranging from 50 to 580 μm. They reported that average compressive strength of scaffolds with a porosity of ~50% reached 50.3 MPa after sintering in a microwave furnace^24^. Li *et al*. produced glass-ceramic scaffolds through a rapid prototyping technique called photosensitive resin mold^25^. The initial composition of scaffold was 45S5 Bioglass® and it converted to a semi-crystalline material after sintering. Sintered scaffolds showed a compressive strength of ~12.4 MPa at 61% porosity. Seol *et al*. aimed to create robust ceramic scaffolds by utilising microstereolithography^26^. They used a ratio of 8:2 (v/v) between photocurable resin and ceramic powder (HA/β-TCP), and the desired 3D structure was fabricated by microstereolithography system with a 450 W ultraviolent lamp. Scaffolds sintered at 1400 °C showed a compressive strength of 2 MPa. They managed to increase compressive strength of scaffolds to 4 MPa by coating its surface with PCL. Swain *et al*. synthesised HA powder with high phase stability at a temperature of 1250 °C. This allowed them to achieve HA scaffolds with dense struts. The resulting HA scaffold with 60% porosity showed a compressive strength of 11 MPa. Tiainen *et al*. used polymer sponge replication method to produce highly porous and strong titanium dioxide scaffolds^27^. To achieve a high compressive strength some of the sintered scaffolds were recoated with the slurry and sintered again at 1500 °C up to 40 h. Single coated scaffolds with 90% porosity showed a compressive strength of ~0.8 MPa. Compressive strength increased to 3.4 MPa for double coated scaffolds (~88% porosity). So far values reported in the literature for fabricated ceramic scaffolds with porosity from 92-50% lie between 0.01 to 80 MPa^28^. The aim of this study was to utilise a direct ink writing method to fabricate glass-ceramic scaffolds with anisotropic structure and distinct pore geometry with required porosity and sufficient mechanical strength for treatment of bone defects under load. Direct ink writing is a method of fabrication that confers materials flexibility, low cost, and freedom to construct arbitrary 3D structures layer by layer^29^. Hence, we designed a scaffold with hexagonal pore geometry to achieve a higher contact area between printed layers, producing a highly anisotropic scaffold architecture leading to enhanced load transfer compared other conventional patterns (rectangular, curved and zigzag)(Fig. 1).

## Results and Discussion

**Figure 1:**
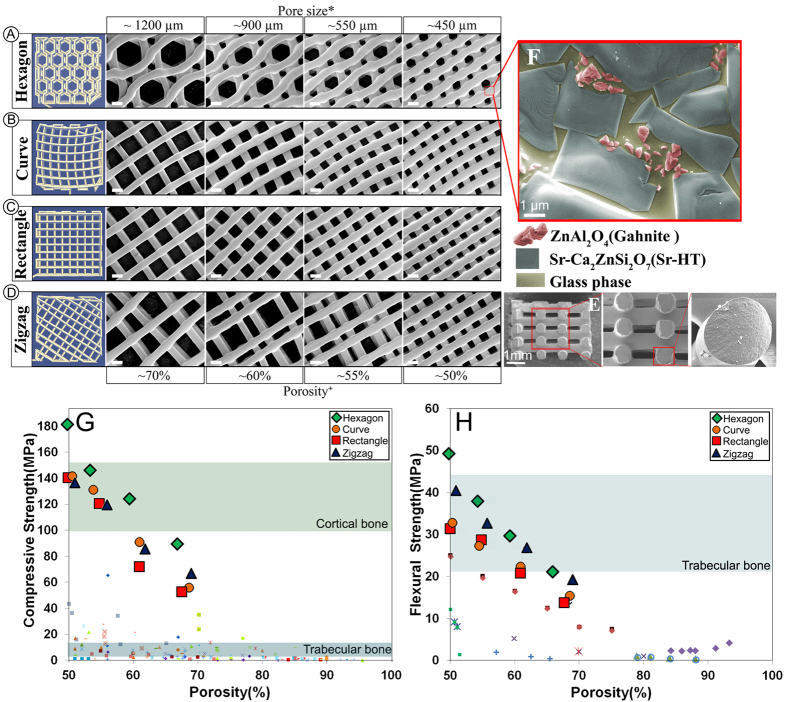
Computer aided design models (left column) and SEM images of examined scaffolds (Scale bars: 500 μm unless stated otherwise). (**a**) Hexagonal, (**b**) Curved, (**c**) Rectangular and (**d**)Zigzag design. (**e**) SEM images of fracture surface of a Sr-HT-Gahnite scaffold prepared by direct ink writing (perpendicular to the deposition plane, z direction), revealing the solid struts without any microporosity in the microstructure. ( **f** ) The microstructure of Sr-HT-Gahnite scaffolds consisting of three phases of (1) Sr-HT grains, (2) ZnAl2O4 crystals and (3) a glass phase between the grains. *Pore sizes calculated from SEM images at XY direction from the average of maximum and minimum distances between layers at intersections (Refer to supplementary table). +Porosity of scaffolds was calculated by Archimedes and micro–computed tomography (μ-CT) and average numbers were rounded to reported values (Refer to supplementary table). (**g**) Compressive strength of Sr-HT-Gahnite scaffolds with distinct pore geometries vs porosity. Comparison with compiled data from literature studies for polymer, composite, bioactive ceramic and glass scaffolds at porosities between 50 and 95%. (**h**) Flexural strength of Sr-HT-Gahnite scaffolds with hydroxyapatite and bioactive glass scaffolds. Each style of point corresponds to a different literature value. Standard deviations from average values are reported in Table 1 at the supplementary information.

Previously, we fabricated a bioactive glass-ceramic, hereafter called Sr-HT Gahnite (Patent #AU2011903923), with a unique triphasic microstructure consisting of (1) strontium (Sr) doped hardystonite (Ca_2_ZnSi_2_O_7_, HT) grains, (2) clusters of submicron gahnite (ZnAl_2_O_4_) crystals and (3) a glass phase (Fig. 1f)^30^,^31^. We demonstrated both *in vitro* and *in vivo* bioactivity of this material. Moreover, we showed that Sr-HT-gahnite microstructure was resistant to crack propagation and a dense structure could be achieved with liquid phase sintering^31^. In the study herein, we fabricated four scaffolds with distinctive pore geometries (rectangular, hexagonal, curved or zigzag) at a variety of porosities (~50, ~55, ~60 and ~70%, the diameter of the deposited struts was held at ~540 μm) by direct ink writing technique (Fig. 1a–d). Rectangular pattern is a conventional design for scaffolds fabricated by direct ink writing method where struts have angle of 90° at the point of intersections (Fig. 1c). Zigzag pattern contained non-parallel struts with varying angles at intersections and pore-size gradients (Fig. 1d). Curved strut is a modified architecture of rectangular pattern which contains a minimum and maximum angle of 70° and 115° between struts at the points of intersection (Fig. 1b). Hexagonal pattern is a unique design with a maximum contact area between layers at intersections while maintaining the high porosity (Fig. 1a). Figure 1 shows SEM images of strut arrangement of Sr-HT-Gahnite scaffolds. There is an increase in strut width by ~60μm at the intersections due to surface tension and binding effects between layers during sintering. This strong binding at intersections facilitates load transfer between the layers of the scaffold. Presence of melt phase at sintering temperature resulted in full densification of the struts with no microporosity at the centre of struts (Fig. 1e). The compressive strength of scaffolds was measured at the perpendicular and parallel direction to the pore channels. The compressive strength perpendicular to the pore channels was approximately one third of that measured in the parallel direction, which reflects the anisotropic structure of the scaffolds and is similar to what has been observed for cortical bone^32^. Figure 1g compares the compressive strength of bioactive glass, ceramic, polymer and composite scaffolds fabricated using conventional techniques to that for our Sr-HT-Gahnite scaffolds (data are compiled from several review articles^5^,^7^,^8^,^28^,^33^,^34^,^35^). The strength parallel to the pore orientation of hexagonal patterned scaffolds reached as high as 139 MPa at a porosity of ~60%. The elastic modulus, determined from the linear region of stress-strain curve, was 2.4 GPa, within the range of that for trabecular bone (0.1–5 GPa). The average strength of the hexagon patterned scaffolds was 122 ± 12 MPa, which is in the range of that for human cortical bone (100–150 MPa). This is 4–5 times the strength for the bioactive glass and hydroxyapatite scaffolds at comparable porosity, pore size and interconnectivity and three orders of magnitude higher than that of values reported for polymeric scaffolds.

**Figure 2:**
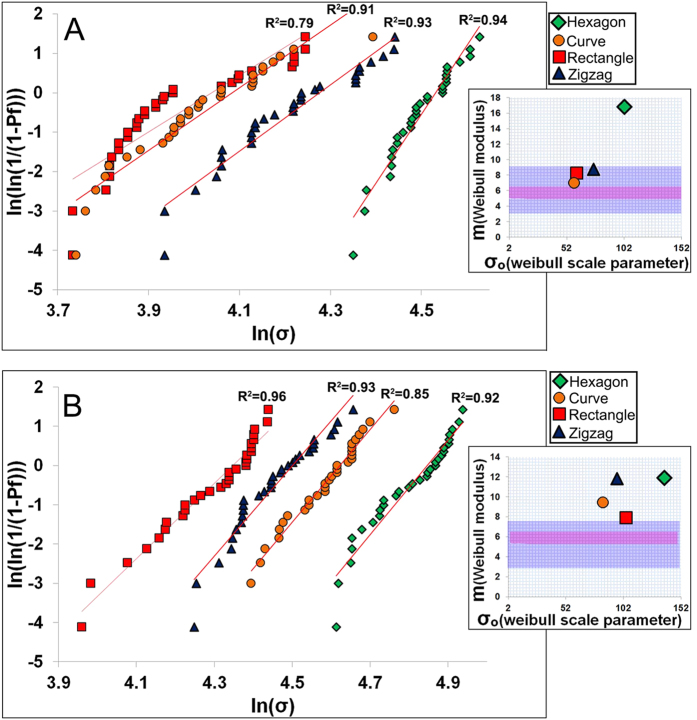
Weibull plots of compressive strength, Weibull modulus (m) and Weibull scale parameter (σ_o_) for Sr-HT-Gahnite scaffolds with (A) ~70% and (B) ~60% porosity. Purple area indicates the Weibull modulus for porous hydroxyapatites and pink area is that for porous bioactive glasses.

Relative to the other pore-shapes, scaffolds with hexagonal patterns showed the highest compressive strength at any given porosity. This increased from 90 MPa at ~70% porosity to 180 MPa at ~50% porosity. This is attributed to higher contact area between printed struts leading to enhanced load transfer as well as the highly anisotropic architecture of hexagonal patterned scaffolds. Others have correlated high strengths to anisotropic architecture of scaffolds. For example, Deville *et al*. fabricated hydroxyapatite scaffolds with a lamellar architecture using freeze casting technique with a compressive strength of 65 MPa at 56% porosity^36^. They showed that lamellar architecture and pore shape anisotropy can lead in unusually high compressive strength for scaffolds. Flexural strength defines resistance of a material to both tensile and compression forces. There is limited published data on the flexural strength of highly porous ceramics and glasses. This is because these materials have been too fragile to withstand the testing procedure including preparation of standard specimens, post-processing and handling. The limited data available, however, shows that although flexural strength of ceramics and glasses decreases with increasing porosity, its dependence on porosity is not as strong as that for compressive strength, particularly at porosities between 90 to 50%. Figure 1h compares flexural strength of calcium phosphate and bioactive glass scaffolds (data are compiled from following reports^37^,^38^,^39^,^40^,^41^,^42^) to Sr-HT-Gahnite scaffolds. In this range of porosity, the flexural strength of hydroxyapatite and bioactive glass scaffolds span from 0.2 to 24 MPa. While this is within the range of cancellous bone (10–25 MPa), it is well below those for cortical bone (135–193 MPa). Flexural strength for Sr-HT-Gahnite scaffolds with hexagonal patterns was significantly higher; ranging from 21 and 51 MPa for porosities between ~70 and ~50%, respectively. Flexural strength values for Sr-HT-Gahnite scaffolds were approximately three times less than those measured for the compressive strength. This is a common behaviour of ceramic and glass materials which is attributed to differences in failure mode in compression and bending. These materials typically fail under compression loading by an accumulation of microcracks that grow parallel to the applied load, whereas under bending loads, a single crack grows perpendicular to the applied load and leads to a catastrophic failure^43^. Fracture of porous ceramic and glass materials follows linear elastic fracture mechanics; the strength of these materials is strongly dependent on the size, distribution and orientation of flaws, which act as stress concentrators^44^, and typically shows a considerable variation between samples. The reliability or the probability of failure occurring from critical flaws in such materials is important when designing materials for use in the regeneration of large bone defects under load. This reliability is quantified by a probability function proposed by Weibull and is given as a cumulative distribution equation (1)^45^:


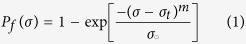


where *P*_*f*_ is the probability of failure at a stress σ, σ_o_ is the Weibull scale parameter (the stress at which the probability of failure is 63%), σ_t_ is the threshold stress below which no failure occurs in the material, which can be taken as zero for ceramics, and m is the Weibull modulus. The Weibull modulus is commonly used as a measure of the mechanical reliability or the probability of failure of ceramic materials. A higher Weibull modulus, m, indicates a narrower distribution in strength and therefore a more reliable material. The *P*_*f*_ can be evaluated using the below equation (2):


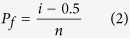


where n is total number of specimens tested and *i* is the specimen rank in ascending order of failure strength. Total number of specimens should not be smaller than 30 to get statistically significant results. Figure 2 shows Weibull plots of the compressive strength data for Sr-HT-Gahnite scaffolds with four patterns at porosities of ~60 and ~70%. Except for scaffolds with rectangular patterns at 70% porosity, the plots were approximately linear over most of the stress range indicating that one type flaw dominated the fracture process. Least squares fitting of a straight line through the date points gave a maximum Weibull modulus of 17 and 12 for hexagonal patterned scaffolds at ~70% and ~60% respectively. Architecture of scaffolds significantly altered the Weibull modulus (m) and scale parameter (σ_o_), as shown in Fig. 2a,b insets. Weibull modulus values changed in a range of 7.1 to 17 for scaffolds with 70% porosity and 7.7 to 12 for scaffolds with 60% porosity. These results demonstrate pore geometry significantly affects the reliability of scaffolds under compression forces. The Weibull modulus of dense or nearly dense ceramics and glasses has been reported to be in the range of 5–20^46^. While there are a limited number of studies on reliability of porous bioactive materials, Liu *et al*. fabricated a silicate bioactive glass with 47% porosity and reported a Weibull modulus of 12 in compression test^46^. Under the same allowable failure probabilities, the Sr-HT-Gahnite scaffolds, particularly those with a hexagonal pattern, showed a failure strength far higher (~5 times) than traditional ceramic and glass scaffolds. Based on Weibull modulus data, when a Sr-HT-Gahnite scaffold with ~70% porosity is subjected to a clinically relevant compressive stress of 11 MPa, then its failure probability is equal to 0.01 (1 in 100 scaffolds is predicted to fail). For calcium phosphate scaffolds with similar porosity, the failure probability is 0.8 (8 scaffolds from 10 are predicted to fail)^47^.

**Figure 3:**
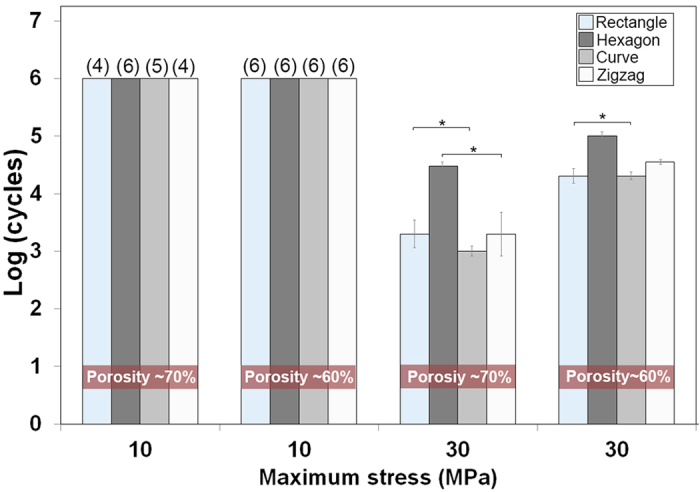
Fatigue life (average number of cycles to failure) of Sr-HT-Gahnite scaffolds under cyclic compressive stress at 60 and 70% porosity. (*Significant difference between groups, p < 0.05). The numbers on top of each bar indicates the number of scaffolds that survived 106 cycles after finishing the test.

One of the crucial, yet commonly neglected, requirements of a scaffold to be used for bone regeneration in load bearing applications is their resistance to cyclic loading (fatigue resistance). Generally materials fail at much lower forces than their nominal strength when subjected to cyclic loading and unloading. Failure of materials by fatigue is associated with adverse body responses, while cyclic loading of scaffolds is reported to result in reduced healing times^2^. Assuming a uniform load distribution, a femoral bone cross-sectional area of ~ 6 cm^2^ and a body weight of 70 Kg, the cyclic stress on an implant in a femoral SBD would be between 5–14 MPa^48^,^49^. To assess their fatigue life, Sr-HT-Gahnite scaffolds (n = 6) between 60 and 70% porosities, were subjected to two cyclic compression loadings (1–10 MPa and 3–30 MPa) at frequency of 5 Hz and fatigue life recorded as the number of stress cycles sustained before catastrophic failure. For cyclic stress of 1–10 MPa, all scaffolds with ~60% porosity withstood 10^6^ cycles (Fig. 3). When the porosity increased to ~70%, only those scaffolds with the hexagonal pattern survived 10^6^ cycles. For rectangular, zigzag and curved patterns only 4, 5 and 4 scaffolds out of 6 scaffolds, respectively, could withstand 10^6^ cycles. As the cyclic stress amplitude was increased to 3–30 MPa, mean fatigue life of scaffolds was decreased to 10^4.8^ (zigzag), 10^4.3^ (curved), 10^5^ (hexagonal) and 10^4.3^ (rectangular) for ~60% porosity and to 10^3.2^ (zigzag), 10^3^ (curved), 10^4.2^ (hexagonal) and 10^3.2^ (rectangular) for 70% porosity. The results demonstrate the compatibility of Sr-HT-Gahnite scaffolds (in particular scaffolds with hexagonal patterns) with conditions equivalent to normal physiological stresses.

**Figure 4:**
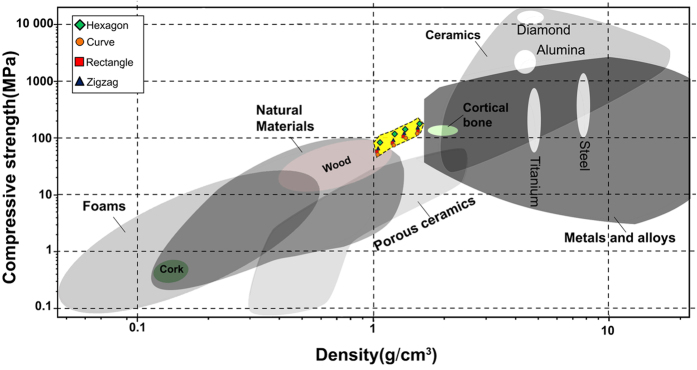
Compressive strength–density Ashby chart showing the compressive strength of Sr-HT-Gahnite scaffolds fabricated by direct ink writing compared with other materials at various densities.

Figure 4 shows the Ashby chart for compressive strength versus density of natural/synthetic materials, including anisotropic Sr-HT-Gahnite scaffolds. Compared with other materials with a density between 1 and 2 g/cm^3^, the Sr-HT-Gahnite scaffolds obtain relatively high values. Hexagonal patterned scaffolds exceed all technical foam materials with specific compressive strength falling in the range of that for cortical bone as well as that for advanced metallic alloys.

In this study, we developed highly porous and strong glass-ceramic scaffolds by direct ink writing method with strength comparable to cortical bone. Through optimisation of pore geometry, we demonstrated a general method to enhance scaffold porosity and mechanical strength. The scaffolds with anisotropic structure particularly those with hexagonal patterns showed a high compressive strength, fatigue resistance, flexural strength and reliability in compression. We were able to achieve properties equivalent to that of cortical bone, in addition to having sufficient porosity to support cellular infiltration and tissue regrowth, compared to those for conventional pore geometry. Our results demonstrate the effective role of pore geometry as a design factor in fabrication of strong scaffolds. These scaffolds have promising applications as synthetic bone substitutes for use in load bearing bone applications.

## Methods

### Materials

Sr-HT(Sr doped Ca_2_ZnSi_2_O_7_(HT)) powder was prepared by sol-gel method^50^ using tetraethyl orthosilicate ((C_2_H_5_O)_4_Si, TEOS), zinc nitrate hexahydrate (Zn(NO_3_)_2_.6H_2_O), calcium nitrate tetrahydrate (Ca(NO_3_)_2_.4H_2_O) and strontium nitrate (Sr(NO_3_)_2_) as raw materials (all materials purchased from Sigma-Aldrich, USA). The TEOS was mixed with water and 2 M HNO_3_ (mol ratio: TEOS/H_2_O/HNO_3_ = 1:8:0.16) and hydrolysed for 30 min under stirring. Then, the Zn(NO_3_)_2_.6 H_2_O, Ca(NO_3_)_2_.4H_2_O and Sr(NO_3_)_2_ (5 wt%) solution were added into the mixture (mol ratio: TEOS/Zn(NO_3_)_2_.6H_2_O/Ca(NO_3_)_2_.4H_2_O = 2:1:2), and reactants were stirred for 5 h at room temperature. After the reaction, the solution was maintained at 60 °C for 1 day and dried at 120 °C for 2 days to obtain the dry gel. The dry gel was calcined at 1200 °C for 3 h. The agglomerated Sr-HT and 15 wt.% of aluminium oxide(Al_2_O_3_, Sigma-Aldirch) powders, zirconia balls (diameter: 20 mm and 1 mm, weight ratio of ball to powder: 8) and ethanol (ratio of ethanol(volume) to powder (weight): 2) were added to zirconia jars attached to a ball mill machine (Retsch PM 400, Germany). The obtained particles after grinding for 3 h at 200 rpm had D_50,_ D_10 and_ D_90_ equals to 1 μm, 2.1 μm and 0.52 μm calculate by a laser diffraction particle size analyser (LA-960 HORIBA, Japan).

We formulated an ink by dispersing precursor ceramic particles in a water-based organic solution. A concentrated ink was created by mixing 45 vol. % of obtained powder (55 vol. % Milli-Q water) with 1 wt. % hydroxypropyl methylcellulose solution (5 w%, F4 M Dow Chemical, USA) and 1 wt. % of an anionic surfactant (sodium polyacrylate, Vanderbilt Minerals, LLC USA). After dispersing the powder, viscoelastic behaviour of ink was achieved by drop wise addition of high molecular weight polyethyleminine solution (10 wt%, branched PEI, MW~25,000, Sigma-Aldrich) at pH of 9. To homogenise the ink, it was placed in a zirconia jar and ball milled for 2 h at 100 rpm by using zirconia balls with diameter of 20 mm then sieved through a 25 μm mesh to minimise the presence of aggregates. The resulting ink had a controllable viscoelastic response, which allowed it to flow through the deposition nozzle without aggregation or filtration of its components.

### Direct Ink Writing of Scaffolds

Sr-HT-Gahnite scaffolds were fabricated by printing the inks through a 600 μm custom-made nozzle using a robotic deposition device (Hyrel 3D, USA). The ink was first loaded into a syringe and then it mounted on the robotic arm. The ink was printed on an oil coated glass substrate (4 mm thickness). The printed scaffolds were easily detached from substrate after air-drying for 24 hours. A controlled-heat treatment was used to decompose the organics and sintering the particles into dense struts. The green samples were heated at 1 °C/min to 450 °C and then densified at 1250 °C for 3 h. Prior to characterisations, surface grinding was conducted on the samples to remove the solid walls and ensure that scaffold ends to be tested were flat and parallel.

### Characterisation

The porosity of the sintered scaffolds was measured using the Archimedes method and Micro-Computed Tomography (Skyscan 1072). The reported porosity is an average of values derived from these methods. Field emission scanning electron microscopy, FE-SEM, (Zeiss Ultra plus, Germany) was used to observe the microstructure of the scaffolds. The samples were sputter-coated with gold and examined at an accelerating voltage of 5 kV. The compressive strength of the scaffolds was tested in directions parallel (clinically relevant position for *in-vivo* defects) and perpendicular to the pore channels at a cross-head speed of 0.5 mm/min. Scaffolds (n = 30) with a dimension of 6 mm × 6 mm × 6 mm were subjected to surface grinding to eliminate edge-effects and to obtain parallel testing surfaces. Subsequently, the free surface of scaffolds was polished by a polishing pad to minimise the presence of micro-cracks. The experiment was stopped when the scaffolds went under catastrophic failure. At least 30 samples were tested to get statically reliable values for Weibull analysis. Three-point bending testing was performed on scaffolds with dimension of 3 mm × 5 mm × 25 mm at a crosshead speed of 0.2 mm min^−1^ using a 1KN load cell. The flexural strength was determined from the equation (3):


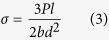


where P is the applied load, l is the outer span, b is the sample width, and d is the thickness of the samples. Fatigue testing of scaffolds was performed in cyclic compression using load control actuation at a frequency of 5 Hz. Two cyclic compressive stresses of 1–10 and 3–30 MPa with minimum to maximum stress ratio of 0.1 was used in this study. Tests continued until failure or 10^6^ cycles were reached. Six samples were tested for each cyclic stress and statistical analysis of the mean fatigue life was performed using one-way analysis of variance (ANOVA) with Tukey’s post hoc test. Differences were considered significant at p < 0.05.

## Additional Information

**How to cite this article**: Roohani-Esfahani, S.-I. *et al*. Design and Fabrication of 3D printed Scaffolds with a Mechanical Strength Comparable to Cortical Bone to Repair Large Bone Defects. *Sci. Rep*. **6**, 19468; doi: 10.1038/srep19468 (2016).

## Supplementary Material

Supplementary Information

Supplementary Movie S1

Supplementary Movie S2

Supplementary Movie S3

Supplementary Movie S4
