# Supplementary material for: Design and Fabrication of 3D printed Scaffolds with a Mechanical Strength Comparable to Cortical Bone to Repair Large Bone Defects
Source: Sci Rep. 2016 Jan 19;6:19468. doi: 10.1038/srep19468 (PMC4726111; doi:10.1038/srep19468)
Supplement: Supplementary Information [file srep19468-s1.pdf]

Design and Fabrication of 3D printed Scaffolds with a Mechanical Strength Comparable to Cortical Bone to Repair Large Bone Defects

Seyed-Iman Roohani-Esfahani[1], Peter Newman[1] and Hala Zreiqat[1]*

[1]Biomaterials and Tissue Engineering Research Unit, School of Aeronautical Mechanical and Mechatronics Engineering, University of Sydney, 2006, NSW, Australia

Supporting Information

Table S1. Numerical results of all tested structures.

Design type	Design No.	Max. pore size(X-Y, μm)	Min. pore size(X-Y, μm)	Pore size (Z, μm)	Porosity,% (Archimedes)	Porosity,% (μ-CT)	Compressive strength(MPa)	Bending strength(MPa)
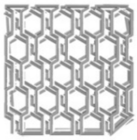	1	1850	350	250	66.5	65.2	90±6	21±3
	2	1258	350	250	58.1	56.7	122±12	27±2
	3	1050	350	250	52.8	51.0	146±14	38±5
	4	638	350	250	45.2	47.8	180±11	50±4
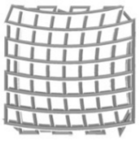	1	1438	-	250	68.5	66.2	55±9	15±1
	2	980	-	250	61.0	57.8	96±9	21±3
	3	578	-	250	55.4	51.2	130±15	28±2
	4	467	-	250	50.5	46.8	145±12	31±7
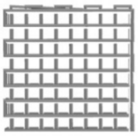	1	1272	-	250	70.0	66.1	53±9	14±2
	2	951	-	250	60.4	61.7	73±9	22±2
	3	652	-	250	55.1	52.1	121±12	28±3
	4	516	-	250	50.1	48.5	140±15	33±5
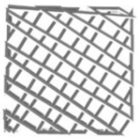	1	1887	566	250	68.3	68.1	68±10	19±2
	2	1413	343	250	63.7	62.3	86±8	27±9
	3	946	157	250	58.1	55.2	118±10	32±6
	4	644	120	250	52.8	49.3	130±17	40±6

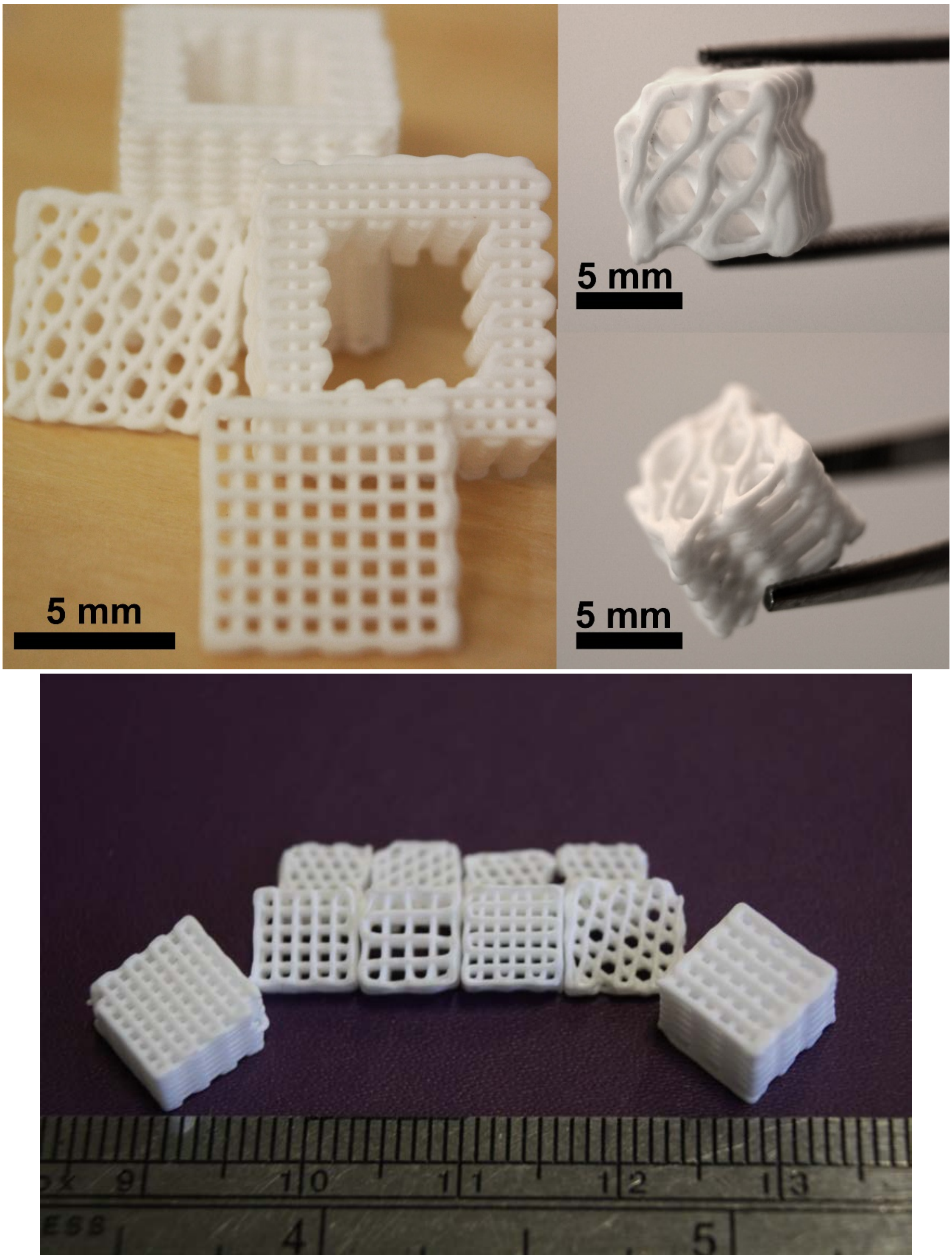

Figure S1. Digital images of printed scaffolds with different patterns and shapes.

Movie S1. Uniaxial compression test of (S1a) hexagonal, (S1b) curved, (S1c) zigzag and (S1d) rectangular patterned scaffold captured by a high-speed camera. “Arrows” show the location of crack initiation and “lines” illustrate the formation of secondary cracks and their

propagation path upon continuous increasing of force. There are more secondary cracks formed in scaffolds with hexagonal pattern before catastrophic failure compared with rectangular and curved designs. This shows that hexagonal pattern facilitates an efficient load transfer in a scaffold by providing a high area of contact at intersections between printed layers.
